# High-mobility group box 1 protein antagonizes the immunosuppressive capacity and therapeutic effect of mesenchymal stem cells in acute kidney injury

**DOI:** 10.1186/s12967-020-02334-8

**Published:** 2020-04-20

**Authors:** Shuo Wang, Songjie Cai, Weitao Zhang, Xigao Liu, Yan Li, Chao Zhang, Yigang Zeng, Ming Xu, Ruiming Rong, Tianshu Yang, Benkang Shi, Anil Chandraker, Cheng Yang, Tongyu Zhu

**Affiliations:** 1 Department of Urology, Zhongshan Hospital, Fudan University, Shanghai Key Laboratory of Organ Transplantation, 180 Fenglin Road, Shanghai, 200032 China; 2 Transplantation Research Center, Renal Division, Brigham and Women’s Hospital, Harvard Medical School, 221 Longwood Ave, LRMC 301, Boston, MA 02115 USA; 3 Department of Urology, Qilu Hospital of Shandong University, Jinan, 250012 China; 4 Shanghai Tenth People’s Hospital, Tongji University School of Medicine, Shanghai, 200072 China; 5 Shanghai Public Health Clinical Center, Fudan University, Shanghai, 201508 China; 6 Fudan Zhangjiang Institute, Shanghai, 201203 China; 7 Department of Transfusion, Zhongshan Hospital, Fudan University, Shanghai, 200032 China

**Keywords:** Mesenchymal stem cell, HMGB1, Ischemia–reperfusion injury, Acute kidney injury, Cell therapy

## Abstract

**Background:**

Kidney ischemia reperfusion injury (IRI) is a common cause of acute kidney injury and an unavoidable consequence of kidney transplantation and still lacks specific therapeutics. Recently, mesenchymal stem cell (MSC) has been emerging as a promising cell-based therapy for IRI in the context of transplantation. MSC negatively regulates the secretion of pro-inflammatory as well as the activation of immune cells during IRI through its unique immunosuppressive property.

**Methods:**

We employed mice kidney IRI model and MSC cell line to monitor the IRI related checkpoints. siRNAs were utilized to knock down the potential key factors for mechanistic analysis. Statistical analysis was performed by using one-way ANOVA with Tukey’s post hoc procedure by SPSS.

**Results:**

The expression of high-mobility group box 1 protein (HMGB1) is increased in the acute phase as well as the recovery stage of IRI. Importantly, the HMGB1 upregulation is correlated with the injury severity. HMGB1 diminishes the MSC induced immunosuppressive capacity in the presence of pro-inflammatory cytokines in vitro. Toll like receptor 4 (TLR4)-mediated inducible nitric oxide synthase (iNOS) inhibition contributes to the negative effect of HMGB1 on MSCs. HMGB1-TLR4 signaling inhibition augments the therapeutic efficacy of MSCs in mice renal IRI model.

**Conclusions:**

These findings demonstrate that HMGB1 plays a crucial role in shaping the immunoregulatory property of MSCs within the microenvironments, providing novel insights into the crosstalk between MSCs and microenvironment components, suggesting HMGB1 signals as a promising target to improve MSC-based therapy.

## Background

Acute kidney injury (AKI) is a common and severe clinical condition with an increasing incidence around the world. It is estimated that the yearly incidence of AKI has exceeded that of myocardial infarction [1]. More severely, AKI is believed to cause approximately 1.7 million deaths per year and contribute to a high risk of development of chronic kidney disease [2]. Due to its high incidence and mortality, AKI remains a critical threat towards public health, and is associated with a substantial socioeconomic burden [3, 4]. The treatment essentially relies on supportive modalities and unfortunately no specific therapeutics, are currently available to treat this disorder.

Mesenchymal stem cells (MSCs), or mesenchymal stromal cells, are adult stem cells originating from the mesoderm [5, 6]. For decades, MSCs have been under intensive investigation as a potential treatment for various diseases including kidney injury [7–9]. Unlike embryonic stem cells, MSCs can be feasibly isolated from a variety of tissues and steadily expanded ex vivo, with minimal ethical issues [10]. Importantly, adoptively transferred MSCs have been shown to home to injured tissues and promote tissue repair, indicating that MSCs are able to provide a site-specific treatment [11–13]. In addition, MSCs are devoid of allogeneic rejections due to its immunoprivileged status [14]. Of note, MSCs possess unique immunoregulatory properties that play a key role in the therapeutic function [15]. These characteristics make MSCs an ideal cell-based therapeutic modality for tissue injuries, inflammatory diseases, and allograft rejections.

Despite the documented therapeutic effects in animal models, MSC-based therapeutic regimens are still not widely applied in clinic [16]. The high plasticity of the immunomodulation of MSCs—the immunosuppressive function of MSCs is regulated by the microenvironments—might result in inconsistencies of the treatment outcomes and hamper clinical application [17, 18]. In this respect, a better understanding of the interplay between MSCs and microenvironmental components is essential to improve the reparative property and clinical potential of MSC-based treatment [19].

High-mobility group box 1 (HMGB1) is a nuclear protein that can be released passively and actively during tissue injuries and other pathological processes [20]. As a classic danger associated molecular pattern (DAMP), HMGB1 is able to exacerbate immune responses, which is presumably encountered by MSCs homing to injured tissues [21]. However, it is not clear whether HMGB1 could also regulate the immunosuppressive effects of MSCs. In the present study, we found that HMGB1 dampens the immunosuppressive capacity of MSC in the presence of inflammatory cytokines in vitro and in vivo. Mechanically, Toll like receptor 4 (TLR4)-mediated inducible nitric oxide synthase (iNOS) inhibition might contribute to the effect of HMGB1 on MSCs. These findings demonstrate that HMGB1 plays a crucial role in shaping the immunoregulatory property of MSCs within the microenvironments, providing novel insights into the crosstalk between MSCs and microenvironment components, suggesting HMGB1 signals as a promising target to improve MSC-based therapy.

## Methods

### Cells and materials

Primary bone marrow-derived MSCs of C57/BL6 mice were purchased from Cyagen Biosciences Inc. (Guangzhou, China). MSCs were cultured in Dulbecco’s modified Eagle’s medium/F12 (DMEM/F12) medium with 10% heat-inactivated fetal bovine serum. The present study used MSCs from 6th to 10th passages. Recombinant human HMGB1 was purchased from Sigma-Aldrich (Shanghai, China) and recombinant murine IFN-γ and TNF-α from PeproTech (Southfield, MI, USA). HMGB1 A and B box were synthesized as previously described. HMGB1 and cytokines were dissolved in PBS.

### Mouse model of renal ischemia-reperfusion injury

This study was approved by the ethics committee of Zhongshan hospital, Fudan University and performed in accordance with established guidelines for the care and use of laboratory animals. C57/BL6 mice (male, 4–6 weeks of age) obtained from Slac Laboratory (Shanghai, China) were used in all experiments. Mice were housed in a pathogen-free, temperature-controlled environment with a 12 h’ light/dark cycle and had free access to food and tap water. To induce an established model of renal IRI, mice were anesthetized with 35 mg/kg intraperitoneal sodium pentobarbital. A midline abdominal incision was made and subsequently bilateral renal pedicles were isolated and clamped for 30 min with microvascular clamps. Reperfusion of both kidneys was confirmed after the removal of the clamps. A sham-operated control group was subjected to similar operations without induction of ischemia. All surgical procedures were conducted on a heated pad to keep the body temperature at 37 °C. Two hours after reperfusion, 1 × 10^6^ MSCs suspended in 120 μl PBS were adoptively transferred into mice via tail vein. Kidney and blood samples of mice were collected for analysis on day 1 post-injury. The number of mice analyzed per group (5 or 6 mice) was chosen to be sufficient to ensure statistical analysis.

### Kidney function

Serum creatinine (Scr) and blood urea nitrogen (BUN) were examined as indicators of renal function by using QuantiChrom Creatinine Assay Kit and QuantiChrom Urea Assay Kit (BioAssay Systems, Hayward, CA, USA) separately.

### Renal histology

Kidney histology was evaluated with formalin-fixed, hematoxylin and eosin (H&E)-stained sections in a blinded manner as described previously. Five different regions of the corticomedullary junction from each mouse was randomly selected and measured. The histological alterations were indicated as ATN score, which was quantified on a 0 to 5 scale according to the percentage of tubular necrosis, tubular dilation and cast formation (0, none; 1, < 11%; 2, 11% to 25%; 3, 26% to 45%; 4, 46% to 75%; 5, > 75%).

### TdT mediated dUTP nick end labeling (TUNEL) assay

To determine the apoptotic magnitude of kidney tissues in response to IRI and MSCs treatment, kidney sections were subjected to TUNEL staining by using In Situ Cell Death Detection Kit (Roche, Indianapolis, USA) according to the manufacturer’s protocol. The number of TUNEL-positive cells was counted under light microscope at × 200 magnification in a blinded manner. Five areas at the corticomedullary junction from each mouse of different groups were assessed.

### Detection of serum cytokines

The concentrations of serum TNF-α, IL-1β, IL-6 and HMGB1 were detected with ELISA kits purchased from R&D Systems (Boston, MA, USA) according to the manufacturer’s instructions.

### T cell proliferation and activation assay

CD4^+^ T cells were purified from spleens of C57BL/6 mice by CD4^+^ T Cell Isolation Kit (Miltenyi Biotec). Freshly isolated T cells were labeled with 2.5 μM CFSE (Invitrogen) and co-cultured with MSCs at different ratios in a Transwell system in the presence of anti-CD3/CD28 microbeads (Invitrogen). After 3 days, T cells were collected and CFSE levels were determined by flow cytometry. For T cell activation detection, CD4^+^ T cells were collected and then incubated with anti-mouse CD4-PE/Cy7 and IFN-γ-PE (Biolegend, SanDiego, CA, USA) for 1 h in dark. The percentage of IFN-γ-positive cells was measured by flow cytometry.

### Lymphocyte infiltration in kidneys

For detection of the lymphocyte infiltration in kidneys, kidney tissues were decapsulated, diced, and digested in collagenase type IV (1 mg/ml, Stemcell Technologies, Vancouver, British Columbia, Canada) for 30 min at 37 °C and then passed through 40 μm Falcon meshes followed by red cell lysis. Renal single cells were washed 3 times with PBS and incubated with anti-mouse CD3-APC, CD4-PE/Cy7 and CD8-PE (Biolegend, SanDiego, CA, USA) for 1 h at 4 °C and detected on FACSCaliber Analyzer (BD Biosciences, San Jose, CA, USA). Data was processed using FlowJo software (Ashland, OR, USA).

### Real-time quantitative polymerase chain reaction (qPCR)

Total RNA was extracted using Trizol reagent (Invitrogen, Carlsbad, USA) according to the manufacturer’s protocol. First-strand cDNA was synthesized using Reverse Transcription Kit (Takara, Dalian, China). Real-time PCR was performed using SYBR Green PCR mix (Roche, Indianapolis, USA) on an ABI Prism^®^ 7500HT Sequence Detection System (Applied Biosystems, CA, USA). The mRNA expression levels of genes of interest were presented as fold exchange that was normalized to β-actin expression in control group. Primer sequences used in the present study are listed in Additional file 2: Table S1.

### Western blotting

Cultured cells were collected and lysed in RIPA buffer containing protease and phosphatase inhibitors on ice to obtain total proteins. Cell protein extracts were electrophoresed on SDS–polyacrylamide gels and then transferred onto nitrocellulose membranes, followed by blocking in 5% nonfat milk for 1 h at room temperature. The membranes were incubated with primary antibodies at indicated dilutions overnight at 4 °C. After incubation with secondary antibodies for 1 h at room temperature, the immunoblot bands were visualized by chemiluminescence and analyzed using ImageJ software.

### Knockdown of RAGE and TLR4 by small interfering RNA (siRNA)

Receptor for advanced glycation end products (RAGE) and TLR4 siRNA were designed and produced by GenePharma (Shanghai, China). RAGE siRNA sequence: sense: GCCAGAAAUUGUGGAUCCUTT; antisense: AGGAUCCACAAUUUCUGGCTT. TLR4 siRNA sequence: sense: GCUAUAGCUUCUCCAAUUUTT; antisense: AAAUUGGAGAAGCUAUAGCTT. Negative control (NC): sense: UUCUCCGAACGUGUCACGUTT; antisense: ACGUGACACGUUCGGAGAATT. The siRNA was transfected into MSCs using Lipofectamine 3000 (Invitrogen) according the manufacturer’s instructions. Briefly, MSCs were subjected to the mixture of siRNA and Lipofectamine 3000 reagent in serum-free DMEM medium for 6 h. And then medium was changed and the cells were harvested for the further experiments.

### Statistical analysis

Data are presented as mean ± standard deviation of replicates. Statistical analysis was performed by using one-way ANOVA with Tukey’s post hoc procedure by SPSS 19.0 software (SPSS Inc., Armonk, NY, USA). A value of *P *< 0.05 was considered statistically significant.

## Results

### HMGB1 expression is upregulated in serum and kidney during renal ischemia–reperfusion injury

**Fig. 1:**
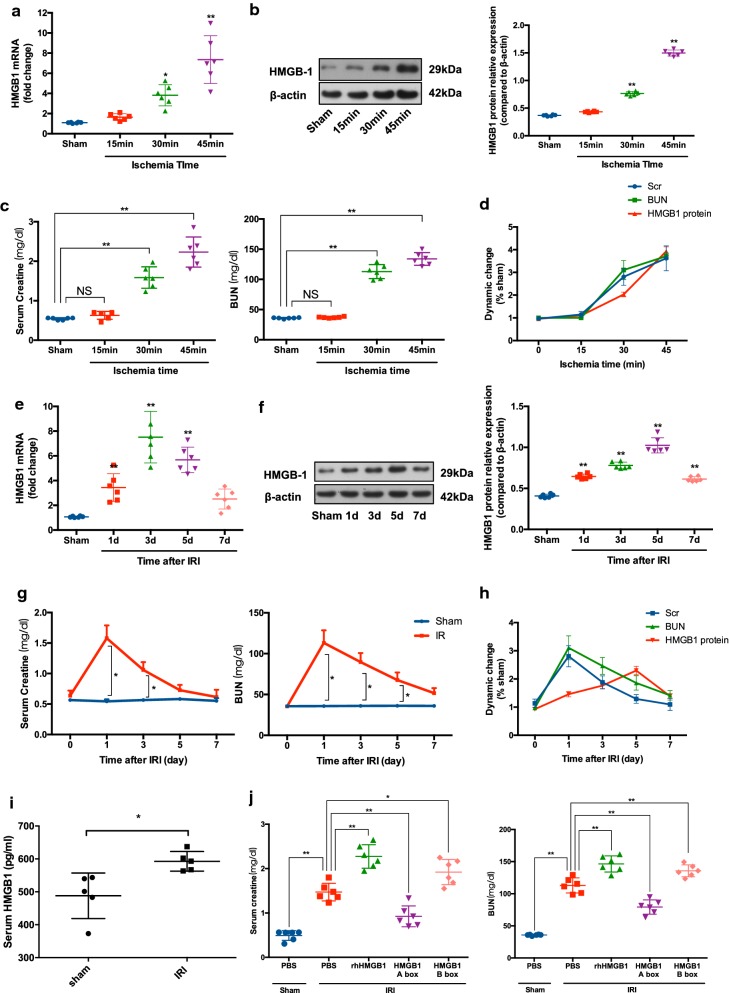
Increased level of HMGB1 in the serum and kidney following IRI. Mice subjected to bilateral renal pedicle clamping for 15, 30 and 45 min were sacrificed at day 1 after reperfusion: Renal mRNA **a** and protein levels **b** of HMGB1 were analyzed by qPCR and western blot analysis separately; **c** Serum levels of creatinine (Scr) and blood urea nitrogen (BUN) were detected; **d** HMGB1 protein, Scr and BUN expressed as a percentage of those parameters for sham groups (n = 6 mice per group). Mice subjected to bilateral renal pedicle clamping for 30 min were sacrificed at the indicated time point: Renal mRNA **e** and protein levels **f** of HMGB1 were analyzed by qPCR and western blot analysis separately; **g** Serum levels of Scr and BUN were detected; **h** HMGB1 protein, Scr and BUN expressed as a percentage of those parameters for sham groups (n = 6 mice per group). **i** Serum level of HMGB1 was determined 24 h after reperfusion with bilateral renal pedicle clamping for 30 min (n = 5 mice per group). **j** Mice were subjected to bilateral renal pedicle clamping for 30 min and then received recombinant HMGB, A box, B box (1 mg/kg body weight) or vehicle PBS by intraperitoneal injection immediately after reperfusion. These mice were sacrificed at day 1 for detection of kidney function (n = 6 mice per group). Data were shown as mean ± SD. *P < 0.05, **P < 0.01

Renal ischemia–reperfusion injury (IRI) is a common reason in prerenal injury. Although it has been reported that HMGB1 is increased during renal IRI, the dynamics of HMGB1 expression after reperfusion is still not fully understood [22–24]. To determine the possible effect of HMGB1 on MSCs within kidneys, we first examine the renal expression of HMGB1 during IRI in the conditions of different ischemia time and different reperfusion time. Postulating that local HMGB1 release might be concomitant with kidney injury, we also measured Scr and BUN levels simultaneously as markers of renal function. As shown in Fig. 1a, b, both HMGB1 mRNA and protein levels increased significantly at day 1 post-IRI with ischemia times of 30 min and 45 min. Figure 1b–d exhibited a similar alteration of Scr and BUN with HMGB1, indicating a positive correlation between HMGB1 release and kidney dysfunction at the early stage of IRI. Furthermore, we subjected mice to bilateral renal pedicles clamping for 30 min and observed the dynamic changes of HMGB1 and kidney function following reperfusion. HMGB1 mRNA and protein were obviously upregulated at day 1 (Fig. 1e, f). Western blot analysis demonstrated that HMGB1 protein continued to increase up to day 5 and remained elevated at day 7. However, in contrast to HMGB1, both Scr and BUN peaked at day 1 and recovered thereafter (Fig. 1g). Importantly, these data suggested that the upregulation of HMGB1 lasts through the early and recovery phase of IRI which is the main therapeutic window of MSC-based treatment for renal IRI (Fig. 1e–h). Moreover, we detected the serum concentration of HMGB1 and found that renal IRI could also result in a significant increase of HMGB1 in serum (Fig. 5i). HMGB1 is a highly-conserved protein and comprises two basic DNA-binding domains (A- and B-box) and an acidic tail. It is known that B-box mediates the main role of HMGB1 and A-box serves as a specific antagonist [25, 26]. To further confirm the destructive effect of HMGB1, mice with renal IRI were subjected to recombinant HMGB1 as well as A box and B box separately. Significant deterioration of kidney function was observed in HMGB1-treated and B box-treated mice compared with the control group. Interestingly, compared with B box, HMGB1 A box protected mice against kidney damage (Fig. 1j). This indicates that kidney IRI is associated with systematic and local increase of HMGB1 which consequently serves as a crucial component of the microenvironment within injured kidneys.

### HMGB1 suppresses the immunosuppressive capacity of MSCs in the presence of pro-inflammatory cytokines

**Fig. 2:**
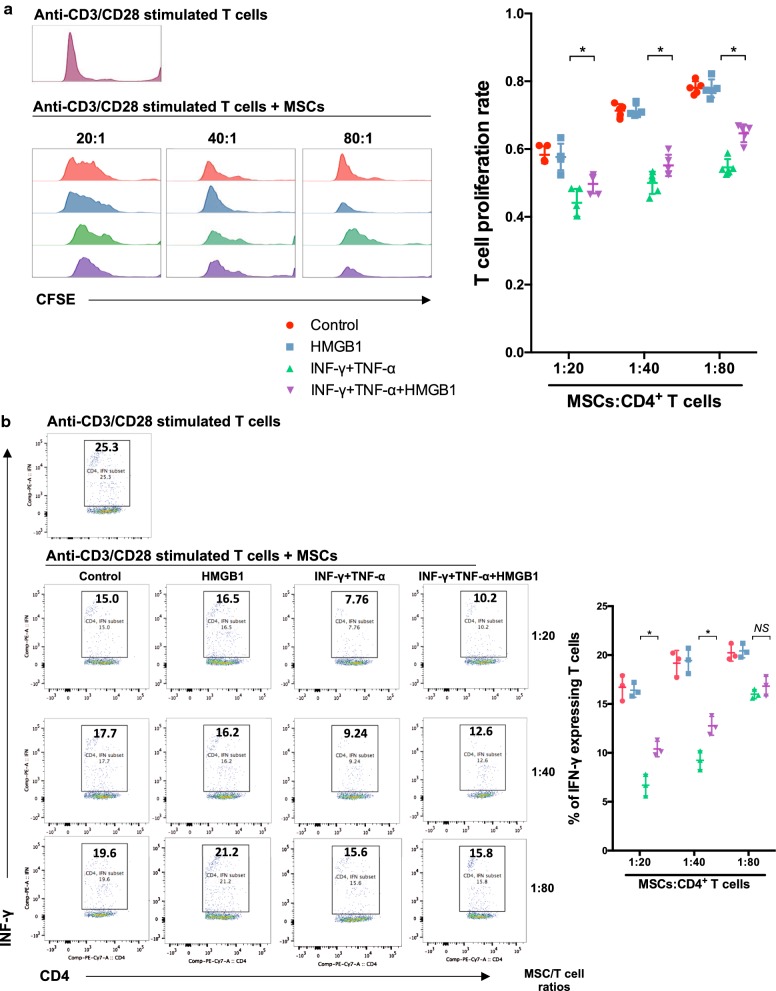
HMGB1 diminished the immunosuppressive effect of MSCs in vitro. MSCs were first treated with or without IFN-γ and TNF-α (5 ng/ml) in the presence or absence of HMGB1 (1 μg/ml) for 24 h, then co-cultured with mice spleen-derived CD4^+^ T cells at ratios of 1:20, 1:40 and 1:80 (MSCs: T cells) for 72 h. **a** T cell proliferation was measured by CFSE assay. **b** The percentage of IFN-γ-positive T cells (gated on CD4^+^ cells) was assessed by flow cytometry to represent the T cell activation. Column scatter graphs were used to visualize the comparison. Each data point represented the average value of triplicated wells from each independent experiment. Data were pooled from at least three independent experiments. Data were shown as mean ± SD. *P < 0.05

As a DAMP, HMGB1 is able to promote inflammation through interaction with a large spectrum of immune cells. However, it is still unclear whether HMGB1 could modulate the immunoregulatory property of MSCs. As we known, T cell proliferation and interferon-γ (IFN-γ) expression experiments are well-documented and widely applied tools to assess the immune response in vivo. We therefore tested the immune regulatory effect of HMGB1-pretreated MSCs with these experiments. CD4^+^ T cells stimulated with anti-CD3 and anti-CD28-coated microbeads were co-cultured with HMGB1-pretreated MSCs in a Transwell system. As expected, a suppression of T cell proliferation by untreated MSCs was observed in CFSE assay (Fig. 2a). Importantly, preconditioning with HMGB1 alone barely showed effect on the immunosuppressive capacity of MSCs. It is prevailingly believed that the combination of interferon-γ (IFN-γ) and tumor necrosis factor-α (TNF-α) is essential to prime naïve MSCs immunoregulatory. Therefore, we further detected the inhibition of T cell proliferation by MSCs pretreated with IFN-γ and TNF-α in the presence or absence of HMGB1. Due to the well-documented pro-inflammatory function of HMGB1, we hypothesized that HMGB1 could augment the immunosuppression of MSCs in a concerted action with pro-inflammatory cytokines. We found that pro-inflammatory cytokines significantly enhanced the immunosuppressive capacity of MSCs on T cell proliferation. Concurrent conditioning with pro-inflammatory cytokines and HMGB1, surprisingly, partially reversed the immune regulatory effect of MSCs compared with pro-inflammatory cytokines-pretreated MSCs. Similarly, addition of HMGB1 could also obviously diminished pro-inflammatory cytokines-induced immunosuppression of MSCs on T cell activation based on intracytoplasmic IFN-γ detection using flow cytometry (Fig. 2b). To verify these observations, we determined T cell proliferation and activation at graded MSC/T cell ratios of 1:20, 1:40 and 1:80. As shown in Fig. 2a, b, a dose-dependent immunosuppression of MSCs was demonstrated and the reversal effect of HMGB1 was confirmed at different ratios. These data demonstrated that HMGB1 impairs the immunosuppressive capacity of MSCs in the presence of pro-inflammatory cytokines.

### HMGB1 diminishes the therapeutic effects of MSCs in mice kidney IRI model

**Fig. 3:**
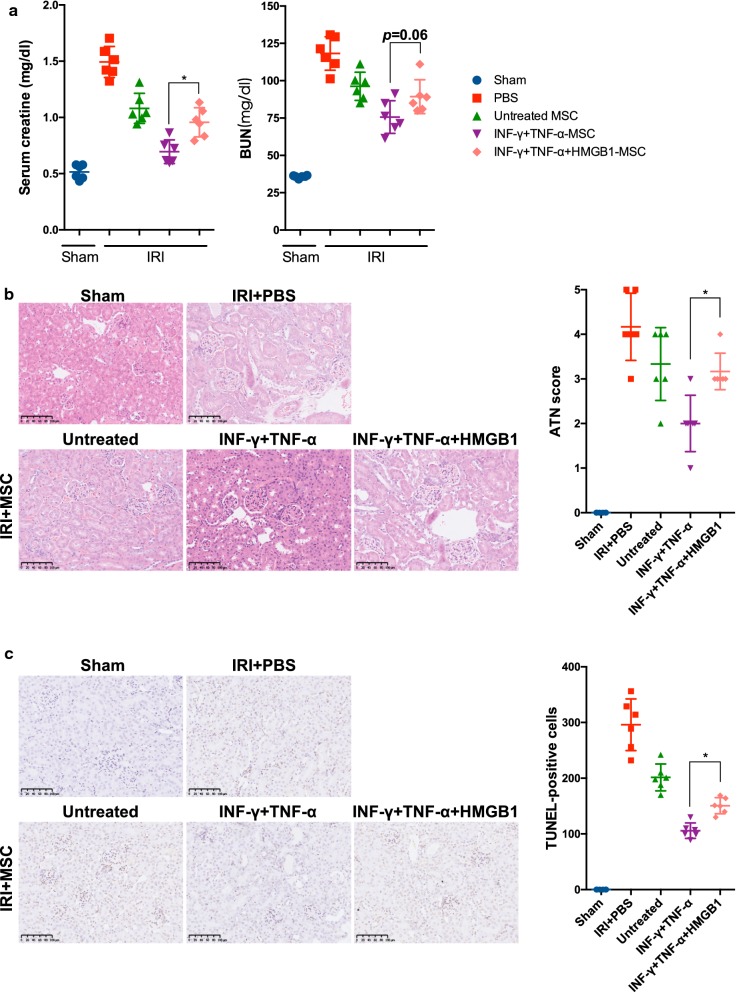
HMGB1 impaired the therapeutic effects of MSCs on renal IRI in mice. Mice were subjected to 30 min bilateral renal IRI and then received differently treated-MSCs (1 × 10^6^) via the tail vein 2 h after reperfusion. Mice were sacrificed at day 1 to collect blood and kidney tissues. **a** Serum levels of Scr and BUN were detected as kidney function. **b** Representative images of H&E staining are exhibited and ATN scores were calculated. **c** Representative photographs of TUNEL staining are demonstrated and quantitative analysis of TUNEL-positive cells was performed. Data were shown as mean ± SD (n = 6 mice per group). *P < 0.05

To fully interpret the impact of HMGB1 on MSCs, we further delineated the therapeutic effect of HMGB1-pretreated MSCs in a murine model of renal IRI. We subjected mice to a regular 30 min bilateral renal IRI and administered MSCs 2 h after reperfusion through the tail vein. MSCs were pre-incubated with and without pro-inflammatory cytokines in the presence or absence of HMGB1 for 24 h. As shown in Fig. 3, IRI caused significant functional damage to the kidneys as expected. Histological analysis showed tubular necrosis, tubular dilatation and cast formation accompanied by increased ATN scores in mice subjected to renal IRI. Compared with vehicle-treated sham control group, IRI also resulted in significant apoptosis within kidneys, exhibited as a dramatic increase in TUNEL-positive cells. In line with previous studies, administration of MSCs significantly reduced the levels of Scr and BUN, improved histological damage and diminished apoptosis [27, 28]. MSCs pre-primed with pro-inflammatory cytokines demonstrated an improvement in therapeutic effect. However, pre-treating MSCs along with HMGB1 could only partially reverse the augmented treatment of MSCs induced by pro-inflammatory cytokines. These data indicate that HMGB1 diminishes the therapeutic effects of MSCs in the presence of inflammation.

### HMGB1 reduces the anti-inflammation effect of MSCs in vivo

**Fig. 4:**
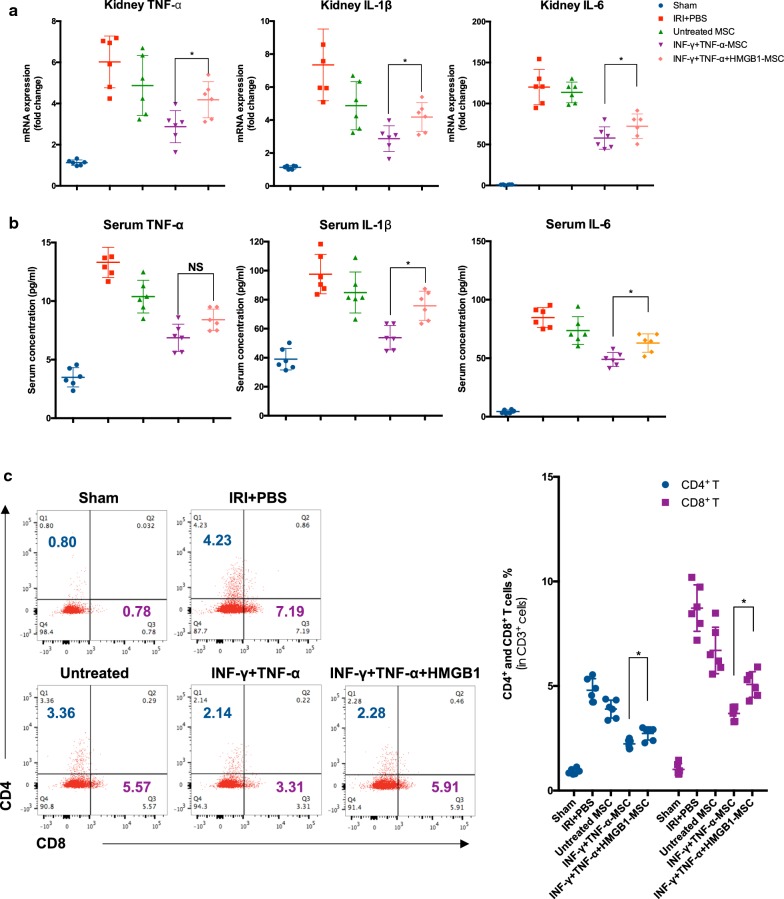
HMGB1 impaired the anti-inflammation efficacy of MSCs in vivo. **a** Renal mRNA levels of TNF-α, IL-1β and IL-6 were analyzed by qPCR. **b** Serum levels of TNF-α, IL-1β and IL-6 were determined by ELISA. **c** Intact kidneys were decapsulated, diced, and digested for single cell suspensions. Renal single cells were then incubated with anti-mouse CD3, CD4 and CD8 antibodies and subsequently analyzed by flow cytometry. Data were shown as mean ± SD (n = 6 mice per group). *P < 0.05

To verify the regulatory effect of HMGB1 on immunosuppression of MSCs in vivo, the inflammatory status during kidney injuries was measured. We first examined the inflammatory cytokine levels in serum and kidney using ELISA and qPCR assay separately. As shown in Fig. 4a, b, the serum concentrations and kidney mRNA levels of TNF-α, IL-1β and IL-6 were dramatically increased due to IRI and treatment with MSCs could indeed alleviate the inflammation. We found, as expected, that pretreatment with pro-inflammatory cytokines enhanced the immunoregulatory ability of MSCs. Importantly, this pro-inflammatory cytokine-induced augmentation of inflammation suppression was obviously reversed by concurrent conditioning with HMGB1. To further evaluate the kidney inflammation, we next detected the lymphocyte infiltration into kidneys using flow cytometry. Similar results were obtained that MSCs treated with a combination of HMGB1 and pro-inflammatory cytokines demonstrated a significant reduction in the inhibition of the extensive lymphocyte infiltration during IRI, compared with MSCs primed by pro-inflammatory cytokines (Fig. 4c). These results were consistent with the histological findings and indicated that conditioning with HMGB1 in the presence of pro-inflammatory cytokines render MSCs less immunosuppressive.

### HMGB1 reverses the pro-inflammatory cytokine-induced iNOS mRNA expression through TLR4

**Fig. 5:**
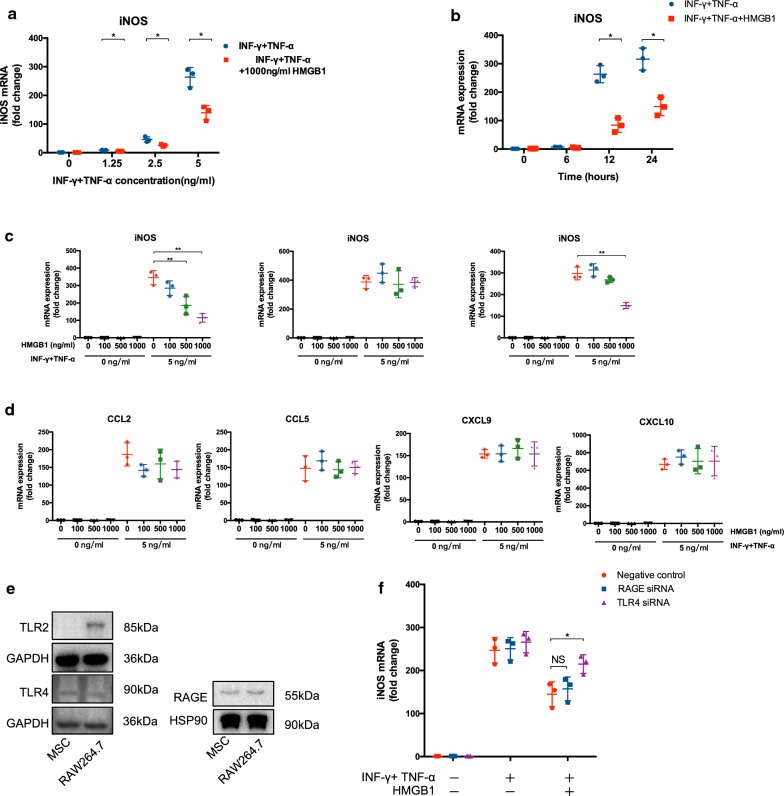
HMGB1 inhibited iNOS mRNA expression via TLR4. **a** MSCs were treated with HMGB1, A box or B box at indicated concentrations for 24 h in the presence or absence of IFN-γ and TNF-α (5 ng/ml). mRNA level of iNOS was analyzed by qPCR. **b** MSCs were treated with IFN-γ and TNF-α at graded concentrations for 24 h in the presence or absence of HMGB1 (1 μg/ml). mRNA level of iNOS was analyzed by qPCR. **c** MSCs were treated with IFN-γ and TNF-α (5 ng/ml) in the presence or absence of HMGB1 (1 μg/ml). mRNA level of iNOS was analyzed by qPCR at indicated time point. **d** MSCs were treated with HMGB1 at indicated concentrations for 24 h in the presence or absence of IFN-γ and TNF-α (5 ng/ml). mRNA level of chemokines was analyzed by qPCR. **e** Receptors were determined by western blot. MSCs were treated with HMGB1 in the presence or absence of IFN-γ and TNF-α. RAGE or TLR4 siRNA were administrated, and iNOS mRNA **f** was examined. Each data point represented the average value of triplicated wells from each independent experiment. Data were pooled from three independent experiments. Data were shown as mean ± SD. *P < 0.05, **P < 0.01

As iNOS plays an indispensable role in mediating the immunosuppressive property of murine MSCs [29], we further determined whether HMGB1 could modulate the expression of iNOS in MSCs in a series of dose–response and time course studies. Under the conditions of different concentrations of IFN-γ and TNF-α (Fig. 5a) as well as different time points (Fig. 5b), HMGB1 could still reduce the iNOS expression in MSCs. As shown in Fig. 5c, iNOS mRNA was indeed inhibited by HMGB1 in a dose-dependent manner in the presence of IFN-γ and TNF-α. Similar results were obtained for B box that serves as the main functional subunit of HMGB1. It has been reported that MSCs, on stimulation with inflamed environments, also produce chemokines which play a synergetic role with immunosuppressive molecules in the immunomodulation of MSCs [29]. In this regard, we detected the mRNA expression of CCL2, CCL5, CXCL9 and CXCL10 in MSCs treated by inflammatory cytokines and HMGB1. Data revealed that HMGB1 had minor effects on these chemokines (Fig. 5d).

Given that HMGB1 has been reported to signal mainly through TLR2, TLR4 and RAGE, the expression of these receptors in MSCs was examined by western blot analysis [30]. Cell expression of all three receptors was confirmed in the Raw 264.7 cell line which acted as a positive control, instead, only RAGE and TLR4 were expressed on MSCs (Fig. 5e). To further determine the role of RAGE and TLR4 on the effect of HMGB1, siRNAs targeting RAGE and TLR4 were used. The knockdown efficacy was demonstrated by western blot (Additional file 1: Fig. S1). Compared with negative control (NC) siRNA and RAGE siRNA, TLR4 siRNA could partially abolish the inhibition of HMGB1 on the iNOS mRNA expression of MSCs (Fig. 5f).

### HMGB1-TLR4 signaling inhibition is a promising strategy for improving MSC-based therapeutic modality

**Fig. 6:**
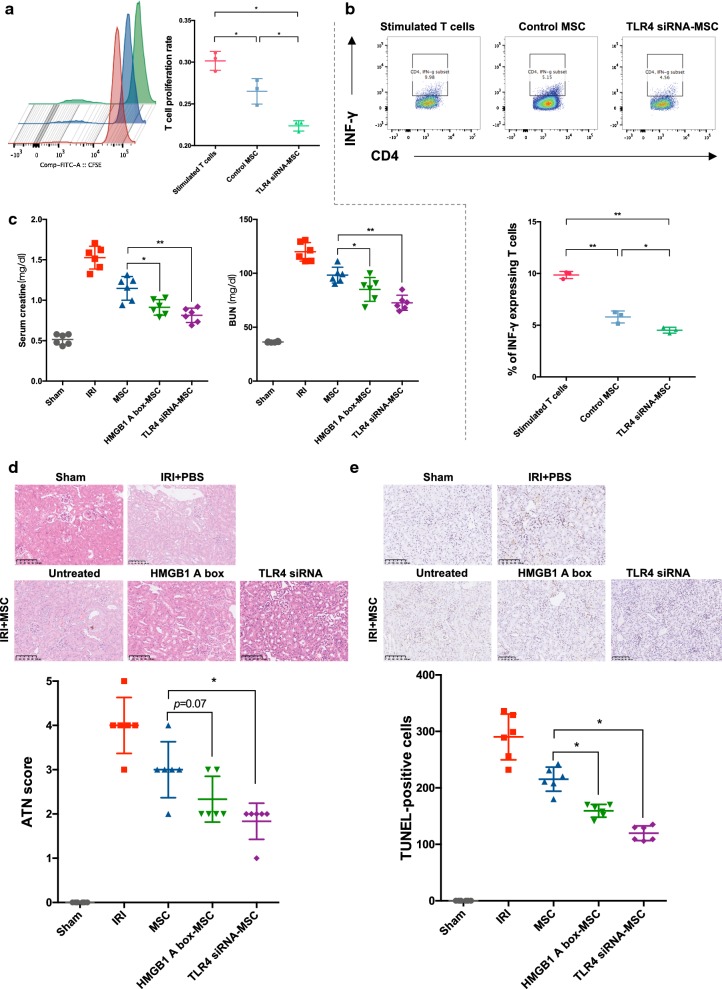
HMGB1-TLR4 blocking enhanced immunosuppression and improved MSC-based therapeutics. TLR4 siRNA-treated MSCs and control MSCs were co-cultured with mice spleen-derived CD4^+^ T cells at a ratio of 1:20 in the presence of IFN-γ, TNF-α (5 ng/ml) and HMGB1 (1 μg/ml) for 72 h. **a** T cell proliferation was measured by CFSE assay. **b** The percentage of IFN-γ expressing T cells was assessed to represent the T cell activation. **c** Differently treated MSCs were administered in mice IRI model. Scr and BUN were detected as indicators of kidney function. **d** Histological analysis was performed to evaluate kidney structure and quantified by ATN scores. **e** TUNEL staining was also conducted to evaluate apoptosis. The number of apoptotic cells was counted under light microscope at × 200 magnification. Each data point represented the average value of triplicated wells from each independent experiment (**a**, **b**). n = 6 mice per group (**c–e**). Data were pooled from at least three independent experiments. Data were shown as mean ± SD. *P < 0.05, **P < 0.01

Within injured kidneys, elevated HMGB1 is proposed to promote inflammatory responses, which indirectly prime MSCs with immunosuppressive properties; on the other hand, it also directly weakens the immunomodulatory efficacy of MSCs through the engagement of TLR4. To circumvent this issue, we adopted a HMGB1-blocking strategy to reinforce the immunosuppression-based therapeutic effect. Before administration in animal models, we repeated T cell proliferation and activation assay in TLR4 siRNA-treated MSCs in the presence of IFN-γ, TNF-α and HMGB1 and we found that TLR4 siRNA enhanced the immunoregulatory function of MSCs (Fig. 6a, b). In a subsequent IRI study, we preconditioned MSCs with HMGB1 A box (a specific antagonist) or TLR4 siRNA to prevent the impact of HMGB1 in the microenvironment within kidneys. As shown in Fig. 6c–e, both strategies improved the reparative efficacy of MSCs.

## Discussion

Recent studies have shown the immunoregulatory effect of MSC in immune disorders, for instance, graft-versus-host disease (GvHD), cardiovascular diseases, liver diseases and autoimmune diseases [31]. In the context of solid organ transplantation, it has been well accepted that the immunosuppressive character of MSC plays a critical role in the reparative effects in IRI [18, 31]. However, the immunosuppression is not an inherent property of MSCs, but adaptively ‘empowered’ by the inflamed microenvironment. Specifically, inflammatory cytokines are required to elicit the immunomodulatory effects of MSCs, among which IFN-γ in the concomitant presence of at least one other cytokines, including TNF-α, IL-1α and IL-1β, is essential [32].

While a permissive microenvironment is essential, the magnitude of the immunosuppressive properties of MSCs is dynamically orchestrated by the surrounding molecular network. It is reported that the pro-inflammatory cytokine IL-17 can enhance the immunosuppressive effect of MSCs by increasing the stability of iNOS mRNA [33]. Conversely, the anti-inflammatory cytokine TGF-β renders MSCs less immunosuppressive through inhibiting iNOS or IDO expression via Smad3 pathways [34]. Likewise, the concurrent addition of IL-10 further diminishes the immunosuppression of MSCs. Unexpectedly, several immunosuppressive drugs including cyclosporine, tacrolimus and dexamethasone that are widely used for organ transplantation and autoimmune disease in clinic routines, can still impair the immunoregulation of MSCs [35–38]. Collectively, it seems like the immunomodulatory factors could paradoxically favor the immune responses towards the opposite side via shaping the bioactivity of MSCs [29, 31]. Despite great progress have been made in this field, the interplay between the MSCs and the components in the microenvironment is not fully delineated.

**Fig. 7:**
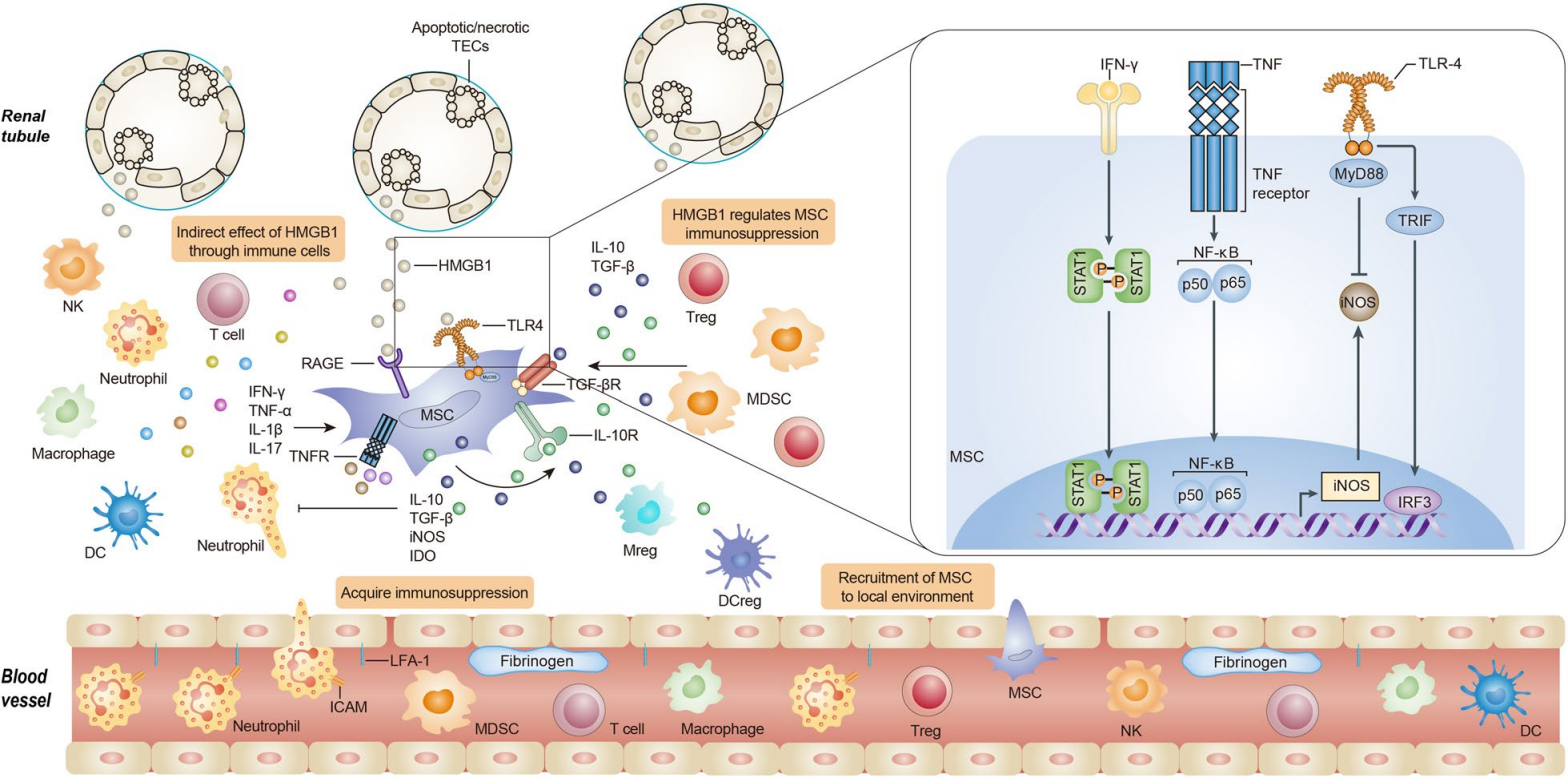
Plasticity of MSCs in immunoregulation: the crosstalk between microenvironments and MSCs. The immunomodulatory function of MSCs relies on the kinds and concentrations of microenvironment components. As a DAMP, HMGB1 plays an important role in initiating and exacerbating immune responses in local environment in response to damages. A subsequent robust pro-inflammatory response renders MSCs immunosuppressive. HMGB1 itself, however, directly reversed the immunosuppression of MSCs through at least in part inhibiting iNOS expression involving TLR4 pathways. Because the treatment efficacy of MSCs is dependent on the immunosuppression to a great extent, HMGB1 can further modulate the reparative effect of MSCs via an integrated action in the microenvironments

DAMPs, in the microenvironment, play a critical role in regulating immune responses, angiogenesis and tissue remodeling [21, 39]. HMGB1 is identified as one of the most important members [20]. Previous studies reported the upregulation of HMGB1 in kidney IRI, kidney transplantation and other kidney injuries [40–42]. In this study, we further found that HMGB1 upregulation is correlated with the injury severity. And we also verified the expression of HMGB1 is increased not only in the acute phase of IRI, but also during the recovery stage. HMGB1 can promote inflammation through pattern recognition receptors expressed on a wide spectrum of both innate and adaptive immune cells. For instance, HMGB1 is able to stimulate dendritic cell maturation and migration, and to mediate the proliferation and cytokine production of T cells [43–46]. According to the theory mentioned above, HMGB1 is proposed to act in synergy with pro-inflammatory cytokines to augment the immunosuppression of MSCs. Ironically, as a key initiator of inflammatory responses, HMGB1 indeed antagonisms the immunosuppressive capacity of MSC ‘licensed’ by the inflamed environments, indicating more complexities in the reciprocity between MSCs and microenvironments than originally appreciated (Fig. 7). Besides HMGB1, DAMPs include S100 proteins, heat-shock proteins and other molecules [39]. It would be of interest to determine whether other DAMPs also influence the immunoregulation of MSCs in the subsequent studies.

As a shared ligand, HMGB1 is believed to induce different cellular reactions through various receptors. In immune cells, generally, HMGB1 induces the signaling pathway to release cytokines when it binds to TLR4, in contrast, HMGB1-RAGE interactions could lead to cell migratory action [30]. With TLR4 and RAGE knockdown by siRNA, we demonstrated that HMGB1 could diminished immunosuppressive iNOS expression in MSCs via TLR4. The engagement of TLR4 on different cells results in cell type-specific responses. Essentially, stimulation of TLR4 initiates myeloid differentiation primary-response protein (MyD88)-dependent and MyD88-independent pathways. MyD88-dependent pathway induces downstream activation of NF-κB which culminates in the production of pro-inflammatory cytokines [47, 48]. Toll/interleukin (IL)-1 receptor domain-containing adaptor-protein inducing interferon-β (TRIF)-mediated signaling is a MyD88-independent pathway that activates the type 1 IFN response through interferon regulatory factor 3 (IRF3). High levels of IRF3 were observed in tumor-associated macrophages with immunoregulatory properties and type 1 IFN can be a substantial anti-inflammatory mediator in certain contexts [49, 50]. In this regard, HMGB1 might reduce MyD88-independent pathway activation or promote MyD88-dependent signaling cascade in inflammatory cytokines-primed MSCs. This perhaps explain the precise molecular mechanisms by which HMGB1 modulate MSC immunosuppression. However, further investigations are still warranted. In recent years, the role of TLR4 in the immunomodulatory capacity of MSCs has been under intensive investigations and inconsistent results are obtained [51]. TLR4 activation has been found to decrease the immunosuppressive function of human bone marrow-derived MSCs through inhibiting jagged 1 expression [52]. In contrast, other group reported the opposite results, although via different mechanisms [53]. Of note, Waterman et al. demonstrated that TLR4 triggering render MSCs a pro-inflammatory phenotype and therefore proposed a dichotomy of MSC1 and MSC2 according to the concept of M1 and M2 macrophages [54]. Adding more uncertainty, some researchers showed no obvious effect of TLR activation on the immunoregulatory function of MSCs [55, 56]. Discrepancies in experimental set-ups may explain, at least in part, the contradictory findings in this field and therefore more investigation is warranted in the future to clarify the contribution of TLR4 to the immune-related activities of MSCs.

To date, most studies regarding the effects of HMGB1 on MSCs have focused on the migration and differentiation capacities. Early research found that HMGB1 potentiates migration [57–60]. On the contrary, recent studies demonstrated apoptotic cells are responsible for the recruitment of MSCs via hepatocyte growth factor (HGF) while HMGB1 released from necrotic cells can reversely inhibit the migration by reducing HGF receptor of MSCs [61, 62]. In terms of differentiation capacities, HMGB1 is reported to facilitate MSC differentiation towards osteoblasts [57, 60, 63, 64], endothelial cells and smooth muscle cells [65]. In addition, HMGB1 can still inhibit the proliferation and regulate the secretion of MSCs [58, 60, 64]. In this scenario, the possible impact of HMGB1 blocking on the biology of MSCs should take into account in subsequent studies.

It is of interest to compare kidney progenitor/stem cells with MSCs. Previous studies have implicated the existence of CD133^+^CD24^+^ renal progenitor/stem cells within adult human kidneys that could differentiate toward the podocyte as well as the tubular lineage [66, 67]. Despite similarities between these cell types in therapeutic potential for renal injury, differences exist in cell-surface markers. Well-accepted markers for MSCs include CD73^+^CD90^+^CD105^+^CD45^−^CD34^−^CD14^−^CD79^−^HLA-DR^−^ [29]. However, due to the paucity of specific markers to study in situ MSCs, our knowledge of the physiological function of endogenous MSCs is limited. Recently, lineage tracing studies have identified GLI1^+^ kidney resident MSCs, that is helpful for further in situ investigations [68].

In this study, we carried out experiments in murine MSCs. It is notable that human MSCs use indoleamine 2, 3-dioxygenase (IDO) but not iNOS as the key immunosuppressive factor [29]. Consistently with our findings, Ramin Lotfi et al. have demonstrated that HMGB1 could decrease IDO expression in human MSCs [69]. Nevertheless, whether HMGB1 has similar effect on human MSCs remains elusive. To address this issue, a separate but more extensive experiment will be designed.

## Conclusions

MSC-based therapy serves as an emerging and promising modality to provide site-specific immunoregulation to treat tissue injuries and inflammation-related disorders. An adequate understanding of the interplay between MSC and the microenvironment is of vital importance to optimize MSC’s therapeutic potential. In this study, we found that HMGB1 could diminish the immunosuppressive capacity of MSC in the presence of pro-inflammatory cytokines in vitro and in vivo. Importantly, we further demonstrated that HMGB1 signaling inhibition strategies would augment the therapeutic effect of MSC on renal IRI. Our research may provide a novel ‘checkpoint’ to improve MSC-based therapies.

## Supplementary information

**Additional file 1: Figure S1.** The knockdown effecacy of TLR4 siRNA and RAGE siRNA.

**Additional file 2: Table S1.** Primers used in qRT-PCR.

## Data Availability

All data generated or analyzed during this study are included in this published article and its additional files.

## Abbreviations

AKI: Acute kidney injury
MSCs: Mesenchymal stem cells
HMGB1: High-mobility group box 1 protein
DAMP: Danger associated molecular pattern
TLR4: Toll like receptor 4
IRI: Ischemia–reperfusion injury
IFN-γ: Interferon-γ
TNF-α: Tumor necrosis factor-α
HGF: Hepatocyte growth factor
iNOS: Inducible nitric oxide synthase
qPCR: Real-time quantitative polymerase chain reaction
TUNEL: TdT mediated dUTP nick end labeling assay
Scr: Serum creatinine
BUN: Blood urea nitrogen
siRNA: Small interfering RNA
RAGE: Receptor for advanced glycation end products
MyD88: Myeloid differentiation primary-response protein
TRIF: Toll/interleukin (IL)-1 receptor domain-containing adaptor-protein inducing interferon-β
IRF3: Interferon regulatory factor 3
