# Supplementary figures and images for: High-mobility group box 1 protein antagonizes the immunosuppressive capacity and therapeutic effect of mesenchymal stem cells in acute kidney injury
Source: J Transl Med. 2020 Apr 20;18:175. doi: 10.1186/s12967-020-02334-8 (PMC7169035; doi:10.1186/s12967-020-02334-8)

Figure S1

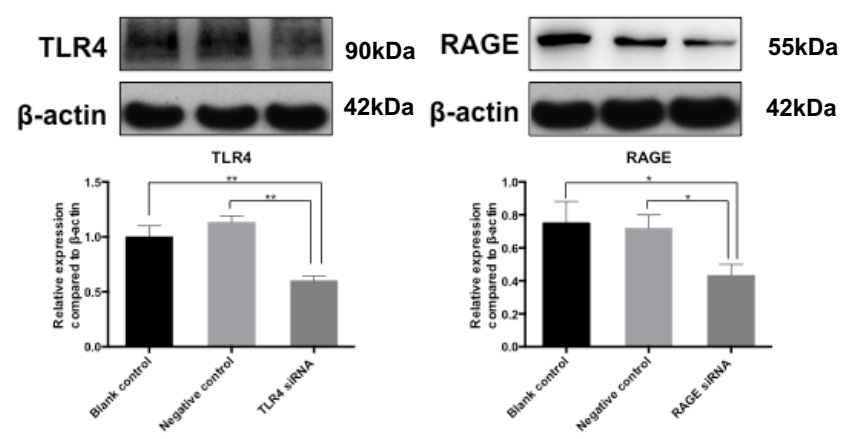

Supplement: Supplementary file 1 — Additional file 1: Figure S1. The knockdown effecacy of TLR4 siRNA and RAGE siRNA. [file 12967_2020_2334_MOESM1_ESM.pdf]
