# Supplementary material for: High-mobility group box 1 protein antagonizes the immunosuppressive capacity and therapeutic effect of mesenchymal stem cells in acute kidney injury
Source: J Transl Med. 2020 Apr 20;18:175. doi: 10.1186/s12967-020-02334-8 (PMC7169035; doi:10.1186/s12967-020-02334-8)
Supplement: Supplementary file 2 — Additional file 2: Table S1. Primers used in qRT-PCR. [file 12967_2020_2334_MOESM2_ESM.pdf]

Table S1: Primers used in qRT-PCR.

Genes	Forward primer	Reverse primer
HMGB1	TGGGCGACTCTGTGCCTC	GCCTCTCGGCTTTTTAGGATC
iNOS	CAGCTGGGCTGTACAAACCTT	CATTGGAAGTGAAGCGTTTCG
TNF-α	TCTTCTCATTCCTGCTTGTGG	GGTCTGGGCCATAGAACTGA
IL-1β	TGTAATGAAAGACGGCACACC	TCTTCTTTGGGTATTGCTTGG
IL-6	GATGGATGCTACCAAACTGGA	CCAGGTAGCTATGGTACTCCAGAA
CCL2	TCTCTCTTCCTCCACCACCATG	GCGTTAACTGCATCTGGCTGA
CCL5	TTTCTACACCAGCAGCAAGTGC	CCTTCGTGTGACAAACACGAC
CXCL9	AGTGTGGAGTTCGAGGAACCCT	TGCAGGAGCATCGTGCATT
CXCL10	TAGCTCAGGCTCGTCAGTTCT	GATGGTGGTTAAGTTCGTGCT
β-actin	CCACGAGCGGTTCCGATG	GCCACAGGATTCCATACCCA
